# Follicle Stimulating Hormone is an accurate predictor of azoospermia in childhood cancer survivors

**DOI:** 10.1371/journal.pone.0181377

**Published:** 2017-07-20

**Authors:** Thomas W. Kelsey, Lauren McConville, Angela B. Edgar, Alex I. Ungurianu, Rod T. Mitchell, Richard A. Anderson, W. Hamish B. Wallace

**Affiliations:** 1 School of Computer Science, University of St. Andrews, St. Andrews, United Kingdom; 2 School of Medicine, University of Edinburgh, Edinburgh, United Kingdom; 3 Department of Haematology/Oncology, Royal Hospital for Sick Children, Edinburgh, United Kingdom; 4 MRC Centre for Reproductive Health, University of Edinburgh, Edinburgh, United Kingdom; University Hospital of Münster, GERMANY

## Abstract

The accuracy of Follicle Stimulating Hormone as a predictor of azoospermia in adult survivors of childhood cancer is unclear, with conflicting results in the published literature. A systematic review and post hoc analysis of combined data (n = 367) were performed on all published studies containing extractable data on both serum Follicle Stimulating Hormone concentration and semen concentration in survivors of childhood cancer. PubMed and Medline databases were searched up to March 2017 by two blind investigators. Articles were included if they contained both serum FSH concentration and semen concentration, used World Health Organisation certified methods for semen analysis, and the study participants were all childhood cancer survivors. There was no evidence for either publication bias or heterogeneity for the five studies. For the combined data (n = 367) the optimal Follicle Stimulating Hormone threshold was 10.4 IU/L with specificity 81% (95% CI 76%–86%) and sensitivity 83% (95% CI 76%–89%). The AUC was 0.89 (95%CI 0.86–0.93). A range of threshold FSH values for the diagnosis of azoospermia with their associated sensitivities and specificities were calculated. This study provides strong supporting evidence for the use of serum Follicle Stimulating Hormone as a surrogate biomarker for azoospermia in adult males who have been treated for childhood cancer.

## Introduction

The potential impact of childhood cancer treatment on male fertility is a significant issue for both families at the time of diagnosis, and the young adult survivor [1, 2]. Treatment at any age, with chemotherapy agents, particularly high doses of alkylating agents, and pelvic radiotherapy, may damage the testes resulting in impaired sperm production[2–7]. While semen analysis remains the gold standard, a serum biomarker of sufficient accuracy, for example Follicle Stimulating Hormone (FSH) would provide a useful indirect assessment of fertility.

The feedback relationship between the seminiferous tubule and the hypothalamus/pituitary underpins the putative value of FSH and inhibin B in the quantitative assessment of spermatogenesis [8]. FSH concentrations are negatively related to sperm concentration in both normal men and in those with testicular dysfunction, whereas serum inhibin B is positively related [9–11]. Both can be used to aid discrimination of obstructive vs non-obstructive azoospermia in infertile men [12] without clear benefit of one over the other, likely reflecting their interdependence and relationship to maturational stages of spermatogenesis [13].

The ready availability and acceptability of serum FSH analysis compared to semen analysis makes it of potential value as a predictor of azoospermia in childhood cancer survivors (CCS), but the literature contains conflicting reports of the sensitivity and specificity of plasma concentrations of FSH in this context. Green et al. [14] found that FSH was unsuitable as predictor of azoospermia in CCS whilst Romerius et al. [15] concluded that FSH was an excellent predictor. It is possible that sources of heterogeneity such as diagnosis, treatment regimens or pubertal status may account for this difference. It is also possible that there is little or no inherent heterogeneity, in which case data can be combined from multiple studies in order to provide a dataset suitable for improved assessment of the true level of diagnostic strength.

In this study we identified studies that have reported FSH and sperm concentrations in CCS, and used them to (a) test the data for homogeneity and (b) to assess the value of FSH as a diagnostic predictor of azoospermia in CCS.

## Materials and methods

Using an established methodology [16–18], a scoping search was carried out using relevant MeSH headings which generated 680 results on PubMed and 973 on Scopus. The Medline search strategy used was **1**. 'Follicle Stimulating Hormone/b **2**. FSH.ti,ab. **3**. Inhibin/bl **4**. Inhibin В/Ы **5**. Follicle stimulating hormone.ti,ab. **6**. exp Sperm Count/ **7**. spermato$.ti,ab. **8**. semen/су **9**. (male adj3 fertil$).ti,ab. **10**. azoospermia.ti,ab. **11**. semen analysis.ti,ab. **12**. sperm concentration.ti, ab. **13**. oligospermia.ti,ab. **14**. semen.ti,ab. **15**. 1 or 2 or 3 or 4 or 5 **16**. 6 or 7 or 8 or 9 or 10 or 11 or 12 or 13 orm14 **17**. 15 and 16 **18**. (HUMANS not ANIMALS).sh. **19**. 17 and 18. Only publications in written in English were screened.

The abstracts of all studies identified were screened, and any studies in cancer survivors that had data on semen analysis and FSH levels were read in full. Studies were selected if they met the following criteria: (i) they contained both serum FSH concentration and semen concentration (either as explicit values or reported in a scatterplot), (ii) World Health Organisation (WHO) certified methods [19] were used in the semen analysis; (ii) the study participants were all childhood cancer survivors, or data was clearly demarcated between childhood cancer survivors and normal controls, in which case only cancer survivor data was extracted; (iii) all study designs were included except case reports. Searches were performed by TWK, LM and WHBW using the PRISMA guidelines for reporting in systematic reviews and meta-analyses [20] between June 2015 and March 2017 (S1 PRISMA checklist). Data extraction was performed by TWK, LM and AIU using version 3.02 of WebPlotDigitizer (http://arohatgi.info/WebPlotDigitizer)); intra- and inter-observer errors were less than 1% for all values, and the extracted datasets closely match the originals in terms of descriptive statistics. Analysis of study quality was performed by LM, RTM and WHBW using the Scottish Intercollegiate Guidelines Network (SIGN) methodology (http://www.sign.ac.uk/assets/sign104_ev_levels.pdf)); all included studies were assessed as 2+ or higher.

In addition to data identified from a systematic search of the literature, we included our own data (S1 Table) used (but not explicitly reported or given as a scatterplot) in a CCS semen quality study [21]. This study involved 33 male survivors of childhood cancer recruited from the oncology database at the Royal Hospital for Sick Children, Edinburgh, from whom FSH levels were obtained in addition to semen concentrations determined according to WHO protocols. For each study participant, we recruited two age-matched controls (n = 66). The volunteers were recruited by means of advertisement in local media and through hospital out-patient clinics, and selected on the basis of the absence of any clinical evidence, on history or physical examination,of reproductive health problems. The Lothian Paediatric and Reproductive Medicine research ethics subcommittee approved the study, and all patients provided written informed consent.

While recognising that different FSH assays were used in the studies included, a detailed comparison has shown ‘fair to strong consistency’ between the relevant assays [22] with most of the variability at the lower end of the normal range thus we have used extracted data without further conversion.

Approval was not required from an ethics committee or institutional review board since our research was limited to use of previously collected, non-identifiable data that has been published in peer reviewed journals which is specifically excluded from Research Ethics Committee review by the National Research Ethics Service guidelines of the UK Health Research Agency [23].

The risk of publication bias was visually assessed by constructing funnels plots, in which calculated diagnostic accuracy is set against statistical precision [24]. In addition, we performed a linear regression of log diagnostic ratios on the inverse root of effective sample sizes as a test for funnel plot asymmetry, where a non-zero slope coefficient is suggestive of significant asymmetry and small study bias [25].

Initial analysis considered the heterogeneity or otherwise of the included studies. This was tested using four distinct techniques: visually by forest plots [26], numerically by calculating the slope of the affine regression equation linking the study diagnostic odds ratios (DOR) to the study thresholds [27, 28] (where a slope close to zero shows homogeneity of the studies), and statistically by (i) calculating the p-value for the chi-squared test of the hypothesis that the studies are heterogeneous (a high p-value suggests homogeneity) and (ii) calculating Higgins I^2^ statistic for measuring inconsistency in meta-analyses [29] (a small value suggests homogeneity). Two statistical tests were used as the interpretation of I^2^ can be misleading, since the importance of inconsistency depends on several factors and the magnitude and direction of effects could lead to a small I^2^ despite a large chi-squared p-value [30].

After combining the data into a single set of (FSH, azoospermic or not azoospermic) pairs, a ROC curve was constructed. 95% confidence intervals for the AUC were calculated using 200 bootstraps of the data set, as were the optimal threshold (i.e. the level of FSH that maximizes the probability of a randomly-selected (azoospermic, not azoospermic) pair from the CCS population being correctly diagnosed) and the 95% confidence intervals for the specificity and sensitivity at each threshold value. All analyses were performed using the *mada* and *pROC* packages for the R statistical language [31].

## Results

**Fig 1:**
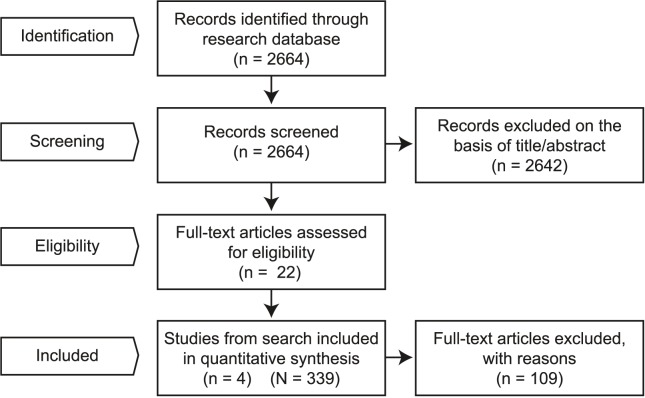
Flow-chart of systematic search methodology. n = number of studies; N = number of childhood cancer survivors fulfilling criteria.

**Fig 2:**
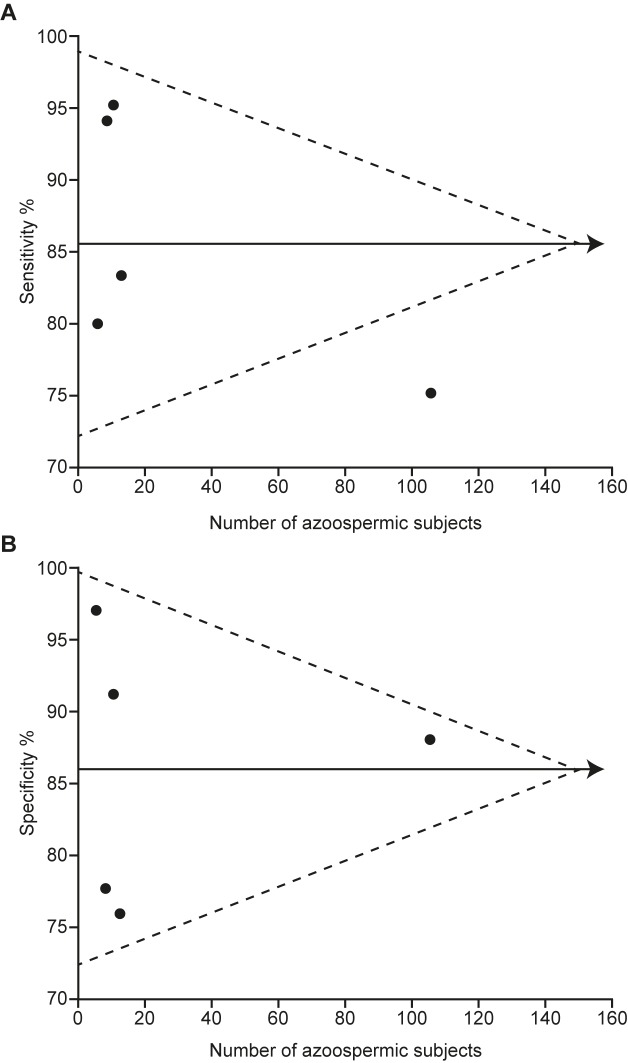
Funnel plots for specificity (upper panel) and sensitivity (lower panel) relating study size to reported diagnostic accuracy for the five studies listed in Table 1. The Chi-squared statistical test for funnel plot asymmetry did not reach statistical significance (p = 0.32 for sensitivity; p = 0.17 for specificity), suggesting a lack of publication bias.

The application of inclusion and exclusion criteria to the studies found in the literature yielded four sources of FSH and semen concentration in CCS (Table 1, S2 Table, Fig 1) [5, 14, 32, 33]. Studies identified for full-text analysis, but excluded are listed in S1 References together with reasons for exclusion. The Chi-squared statistical test for funnel plot asymmetry (Fig 2) did not reach statistical significance (p = 0.32 for sensitivity; p = 0.17 for specificity), suggesting that neither studies with small sample size nor studies with results lacking statistical significance are missing from the literature. As all the included studies used WHO protocols, we conclude that they are at low risk of bias and have low concern about applicability, as specified by the QUODAS-2 and STARD frameworks for reporting diagnostic accuracy [34, 35].

**Table 1:** Characteristics of the included studies.

1^st^ Author	Year	PubMed ID	Number CSS	Age (years, median & range)
Green	2013	23423746	257	30.5, 19.7–59.1
Lähteenmäki	2008	18430073	23	20.5, 15.6–31.2
Rendtorff	2012	21726269	37	25, 19–45
van Beek	2007	17981817	17	27, 17.7–42.6
Thomson	2002	12241775	33	21.9, 16.5–35.2

**Fig 3:**
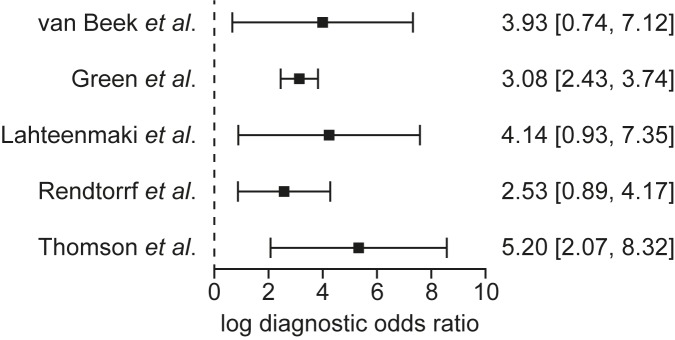
Forest plot of 95% confidence limits for the log-adjusted diagnostic odds ratio for the five studies listed in Table 1. The vertical dashed line denotes the line of no effect. Visual inspection shows that each study is statistically significant in its own right, that the intervals overlap to a great extent, and that therefore the studies are unlikely to be heterogeneous.

The confidence intervals for the log-adjusted DOR for each study have similar ranges, suggesting a lack of significant study heterogeneity (Fig 3). Visual inspection shows that each study is statistically significant in its own right, that the intervals overlap to a great extent, and that therefore the studies are unlikely to be heterogeneous. The slope of the regression equation linking the study log DOR to the study FSH thresholds was close to zero (slope = -0.01), providing numerical evidence for study homogeneity. The chi-squared p-values were 0.32 for study sensitivity and 0.17 for study specificity, supplying no statistically significant evidence for the hypothesis that the studies are heterogeneous. The Higgin’s I^2^ statistic was 0%, the lowest possible indication of study heterogeneity. Taken together, and in conjunction with the lack of publication bias, we conclude that the studies are homogeneous in terms of dependency on FSH thresholds to determine diagnostic accuracy, and hence that combining the study data into a single set results in a representative sample of the CCS population in terms of FSH levels and sperm concentrations.

**Fig 4:**
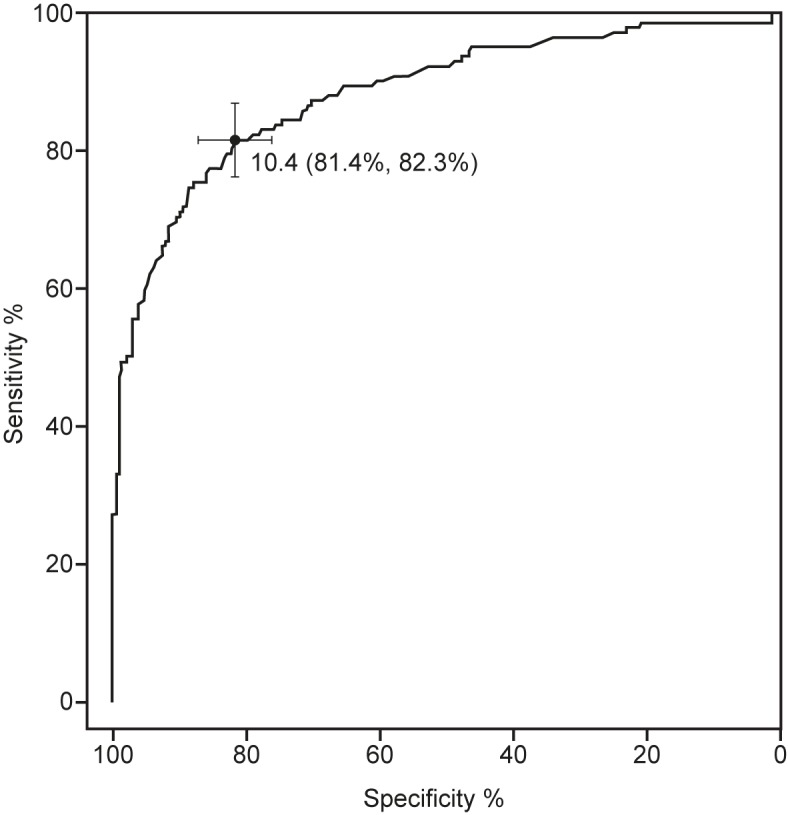
Receiver-operator characteristic (ROC) curve analysis of FSH as predictor of azoospermia (combined cohort: n = 367). Area under the curve: 0·89 (95% CI 0·85–0·92. The optimal diagnostic threshold is 10.4 mIU/mL, with sensitivity 0.814 and specificity 0.823.

For the combined data (n = 367, SI 1, SI 2) the optimal FSH threshold was 10.4 IU/L with specificity 81% (95% CI 76%–86%) and sensitivity 82% (95% CI 76%–88%). The AUC was 0.89 (95%CI 0.85–0.92), demonstrating that FSH is a strong predictor of azoospermia for CCS (Fig 4).

The optimal threshold maximizes the chance of a correct classification for an arbitrary survivor of childhood cancer. In order to quantify FSH levels that minimize misdiagnosis of azoospermia, a range of threshold FSH values for the diagnosis of azoospermia were calculated, together with the median and 95% confidence intervals for their associated sensitivities and specificities (Table 2). A diagnostic threshold of 17 IU/L for FSH gives 94% probability of avoiding misdiagnosis of azoospermia, with 95% confidence interval 90–97% (Table 2).

**Table 2:** Sensitivity and specificity of FSH-based azoospermia diagnosis for a range of threshold values. Median and 95% CI are calculated from 2,000 stratified bootstrap replicates of the combined data (n = 367).

Threshold FSH (IU/L)	Specificity Median	Specificity 95% CI	Sensitivity Median	Sensitivity 95% CI
9	0.743	0.690–0.801	0.851	0.787–0.901
10	0.783	0.730–0.836	0.830	0.766–0.894
10.4	0.814	0.761–0.863	0.823	0.759–0.897
11	0.827	0.774–0.872	0.801	0.731–0.865
12	0.858	0.810–0.903	0.773	0.702–0.837
13	0.885	0.845–0.925	0.752	0.681–0.823
14	0.898	0.858–0.938	0.716	0.638–0.780
15	0.916	0.881–0.951	0.695	0.617–0.766
16	0.925	0.889–0.956	0.660	0.582–0.731
17	0.938	0.903–0.969	0.638	0.560–0.723
18	0.947	0.916–0.974	0.610	0.532–0.688
19	0.951	0.920–0.978	0.589	0.511–0.674
20	0.969	0.943–0.991	0.553	0.468–0.638

## Discussion

We have shown that FSH has strong diagnostic power, with 89% probability that FSH levels will correctly classify as azoospermic, not azoospermic a randomly chosen survivor of childhood cancer (i.e. positive predictive value) [36], with 95% confidence that this probability is within 85% and 92% (Fig 4). For the combined data (n = 367) the optimal Follicle Stimulating Hormone threshold was 10.4 IU/L with specificity 81% (95% CI 76%–86%) and sensitivity 83% (95% CI 76%–89%). The AUC was 0.89 (95%CI 0.86–0.93). This study provides strong supporting evidence for the use of serum Follicle Stimulating Hormone as a surrogate biomarker for azoospermia in adult males who have been treated for childhood cancer. We have also calculated clinically-useful diagnostic levels for a range of FSH thresholds (Table 2).

We have assessed heterogeneity of existing studies using visual, numeric modelling and two distinct statistical tests; none of these suggested any important level of heterogeneity (Figs 2 and 3). This result is of clinical and biomedical interest in its own right, but also allows us safely to combine the data into a single set, which has greater power for statistical analysis than any single study reported to date. While different FSH assays were used in the studies included in this analysis, there is good concordance between them [22].

FSH, inhibin B, and more recently anti-Mullerian hormone have been previously investigated as biomarkers of seminiferous tubule function, often to attempt to predict the surgical recovery of sperm in azoospermic men [12]. The latter two are products of the Sertoli cell, with potentially an additional contribution to serum inhibin B from germ cells [37]. In the post-chemotherapy testis, the key pathology determining azoospermia or not is the presence or absence of spermatogonial stem cells at the end of treatment. This differs therefore from the situation in the more general male infertility population, where disorders of spermatogenic maturation are relatively common, with likely impact on the germ cell-Sertoli cell interaction and production of inhibin B, and feedback regulation of FSH. It is thus possible that serum biomarkers of spermatogenesis may be more accurate in post-chemotherapy assessment than with the wide range of pathologies seen in the general infertile population. We considered the potential value of including these biomarkers in a joint model, but there were insufficient data to do this at present.

Our calculated optimal FSH threshold for classifying a CCS as azoospermic is 10.4 UI/L, where optimal means providing the best tradeoff between sensitivity (i.e. minimized prediction of non-zero sperm concentration for CCS who are in reality azoospermic) and specificity (i.e. minimized prediction of azoospermia for CCS who in reality have non-zero sperm concentration). In clinical practice of long-term follow up of CCS, however, we suggest that a more conservative threshold is more appropriate, since a wrong diagnosis of azoospermia is worse than a false negative. It should also be emphasized that in some azoospermic CCS it is possible to obtain sperm by micro-TESE [38]. The bootstrap sampling used to provide the optimal threshold (necessarily) allows calculation of confidence intervals for sensitivities and specificities for all potential thresholds, and from these we observe that a diagnostic threshold of 17 IU/L for FSH has a 94% probability of avoiding this misdiagnosis, with 95% confidence interval of 90%–97% (Table 2). Our specificity results are in quantitative agreement with a study that reported mean FSH of 22 IU/L in 21 azoospermic CSS compared to 9 IU/L in 10 controls with 81% specificity at a 10 IU/L cutoff [39] compared to our value of 78%. However our calculated sensitivity at this cutoff is higher: 56% [39] compared to 83%.

Serum assessment of FSH is therefore a useful test before the patient is ready to submit a semen sample for analysis, and the present analysis indicates high predictive accuracy. Attempts to survey CCS with universal semen analysis have demonstrated the reluctance of these patients to submit semen samples. In contrast, a blood test is less intrusive and more acceptable to these young CCS [32]. The use of hormone measurement in dried blood spots sent by post has recently been evaluated in the analysis of reproductive function in female cancer survivors [40], and this technique has clear potential to be useful in the male case.

This study provides strong supporting evidence for the use of serum FSH as a useful surrogate biomarker for spermatogenesis in adult males who have been treated for childhood cancer, however semen analysis should always be encouraged and remains the gold standard test of spermatogenesis.

## Supporting information

S1 PRISMA checklist The checklist component of the PRISMA statement. (DOC)

S1 Table Our own data. (XLS)

S2 Table Data from published studies. (XLS)

S1 References Full-text studies excluded, together with reasons for exclusion. (PDF)

## References

[1] Anderson RA, Mitchell RT, Kelsey TW, Spears N, Telfer EE, Wallace WH. Cancer treatment and gonadal function: experimental and established strategies for fertility preservation in children and young adults. The lancet Diabetes & endocrinology. 2015;3(7):556–67. doi: 10.1016/S2213-8587(15)00039-X 25873571 10.1016/S2213-8587(15)00039-X

[2] Skinner R, Mulder RL, Kremer LC, Hudson MM, Constine LS, Bardi E, et al Recommendations for gonadotoxicity surveillance in male childhood, adolescent, and young adult cancer survivors: a report from the International Late Effects of Childhood Cancer Guideline Harmonization Group in collaboration with the PanCareSurFup Consortium. The Lancet Oncology. 2017;18(2):e75–e90. doi: 10.1016/S1470-2045(17)30026-8 28214419 10.1016/S1470-2045(17)30026-8

[3] Greenfield DM, Walters SJ, Coleman RE, Hancock BW, Eastell R, Davies HA, et al Prevalence and consequences of androgen deficiency in young male cancer survivors in a controlled cross-sectional study. The Journal of clinical endocrinology and metabolism. 2007;92(9):3476–82. doi: 10.1210/jc.2006-2744 17579201 10.1210/jc.2006-2744

[4] Greenfield DM, Walters SJ, Coleman RE, Hancock BW, Snowden JA, Shalet SM, et al Quality of life, self-esteem, fatigue, and sexual function in young men after cancer: a controlled cross-sectional study. Cancer. 2010;116(6):1592–601. doi: 10.1002/cncr.24898 20186765 10.1002/cncr.24898

[5] van Beek RD, Smit M, van den Heuvel-Eibrink MM, de Jong FH, Hakvoort-Cammel FG, van den Bos C, et al Inhibin B is superior to FSH as a serum marker for spermatogenesis in men treated for Hodgkin's lymphoma with chemotherapy during childhood. Human reproduction. 2007;22(12):3215–22. doi: 10.1093/humrep/dem313 17981817 10.1093/humrep/dem313

[6] Green DM, Liu W, Kutteh WH, Ke RW, Shelton KC, Sklar CA, et al Cumulative alkylating agent exposure and semen parameters in adult survivors of childhood cancer: a report from the St Jude Lifetime Cohort Study. The Lancet Oncology. 2014;15(11):1215–23. doi: 10.1016/S1470-2045(14)70408-5 25239573 10.1016/S1470-2045(14)70408-5 PMC4192599

[7] Chow EJ, Stratton KL, Leisenring WM, Oeffinger KC, Sklar CA, Donaldson SS, et al Pregnancy after chemotherapy in male and female survivors of childhood cancer treated between 1970 and 1999: a report from the Childhood Cancer Survivor Study cohort. The Lancet Oncology. 2016;17(5):567–76. doi: 10.1016/S1470-2045(16)00086-3 27020005 10.1016/S1470-2045(16)00086-3 PMC4907859

[8] McCullagh DR. Dual Endocrine Activity of the Testes. Science. 1932;76(1957):19–20. doi: 10.1126/science.76.1957.19 17815236 10.1126/science.76.1957.19

[9] Anderson RA, Wallace EM, Groome NP, Bellis AJ, Wu FC. Physiological relationships between inhibin B, follicle stimulating hormone secretion and spermatogenesis in normal men and response to gonadotrophin suppression by exogenous testosterone. Human reproduction. 1997;12(4):746–51. 9159436 10.1093/humrep/12.4.746

[10] Illingworth PJ, Groome NP, Byrd W, Rainey WE, McNeilly AS, Mather JP, et al Inhibin-B: a likely candidate for the physiologically important form of inhibin in men. The Journal of clinical endocrinology and metabolism. 1996;81(4):1321–5. doi: 10.1210/jcem.81.4.8636325 8636325 10.1210/jcem.81.4.8636325

[11] Jensen TK, Andersson AM, Hjollund NH, Scheike T, Kolstad H, Giwercman A, et al Inhibin B as a serum marker of spermatogenesis: correlation to differences in sperm concentration and follicle-stimulating hormone levels. A study of 349 Danish men. The Journal of clinical endocrinology and metabolism. 1997;82(12):4059–63. doi: 10.1210/jcem.82.12.4456 9398713 10.1210/jcem.82.12.4456

[12] Toulis KA, Iliadou PK, Venetis CA, Tsametis C, Tarlatzis BC, Papadimas I, et al Inhibin B and anti-Mullerian hormone as markers of persistent spermatogenesis in men with non-obstructive azoospermia: a meta-analysis of diagnostic accuracy studies. Human reproduction update. 2010;16(6):713–24. doi: 10.1093/humupd/dmq024 20601364 10.1093/humupd/dmq024

[13] Okuma Y, O'Connor AE, Hayashi T, Loveland KL, de Kretser DM, Hedger MP. Regulated production of activin A and inhibin B throughout the cycle of the seminiferous epithelium in the rat. The Journal of endocrinology. 2006;190(2):331–40. doi: 10.1677/joe.1.06706 16899566 10.1677/joe.1.06706

[14] Green DM, Zhu L, Zhang N, Sklar CA, Ke RW, Kutteh WH, et al Lack of specificity of plasma concentrations of inhibin B and follicle-stimulating hormone for identification of azoospermic survivors of childhood cancer: a report from the St Jude lifetime cohort study. Journal of clinical oncology: official journal of the American Society of Clinical Oncology. 2013;31(10):1324–8. doi: 10.1200/JCO.2012.43.7038 23423746 10.1200/JCO.2012.43.7038 PMC3607671

[15] Romerius P, Stahl O, Moell C, Relander T, Cavallin-Stahl E, Wiebe T, et al High risk of azoospermia in men treated for childhood cancer. International journal of andrology. 2011;34(1):69–76. doi: 10.1111/j.1365-2605.2010.01058.x 20345878 10.1111/j.1365-2605.2010.01058.x

[16] Iliodromiti S, Kelsey TW, Anderson RA, Nelson SM. Can anti-Mullerian hormone predict the diagnosis of polycystic ovary syndrome? A systematic review and meta-analysis of extracted data. The Journal of clinical endocrinology and metabolism. 2013;98(8):3332–40. doi: 10.1210/jc.2013-1393 23775353 10.1210/jc.2013-1393

[17] Iliodromiti S, Sassarini J, Kelsey TW, Lindsay RS, Sattar N, Nelson SM. Accuracy of circulating adiponectin for predicting gestational diabetes: a systematic review and meta-analysis. Diabetologia. 2016;59(4):692–9. doi: 10.1007/s00125-015-3855-6 26768001 10.1007/s00125-015-3855-6 PMC4779132

[18] Kelsey TW, Dodwell SK, Wilkinson AG, Greve T, Andersen CY, Anderson RA, et al Ovarian volume throughout life: a validated normative model. PloS one. 2013;8(9):e71465 doi: 10.1371/journal.pone.0071465 24019863 10.1371/journal.pone.0071465 PMC3760857

[19] World Health Organisation. Examination and processing of human semen. 5th ed: The World Health Organisation; 2010.

[20] Moher D, Liberati A, Tetzlaff J, Altman DG, Group P. Preferred reporting items for systematic reviews and meta-analyses: the PRISMA statement. Bmj. 2009;339:b2535 doi: 10.1136/bmj.b2535 19622551 10.1136/bmj.b2535 PMC2714657

[21] Thomson AB, Campbell AJ, Irvine DC, Anderson RA, Kelnar CJ, Wallace WH. Semen quality and spermatozoal DNA integrity in survivors of childhood cancer: a case-control study. Lancet. 2002;360(9330):361–7. 12241775 10.1016/s0140-6736(02)09606-x

[22] Radicioni A, Lenzi A, Spaziani M, Anzuini A, Ruga G, Papi G, et al A multicenter evaluation of immunoassays for follicle-stimulating hormone, luteinizing hormone and testosterone: concordance, imprecision and reference values. Journal of endocrinological investigation. 2013;36(9):739–44. doi: 10.1007/BF03347112 24196213 10.1007/BF03347112

[23] HRA. Does my project require review by a Research Ethics Committee? 2013. Available from: http://www.hra.nhs.uk/documents/2013/09/does-my-project-require-rec-review.pdf.

[24] Sterne JA, Sutton AJ, Ioannidis JP, Terrin N, Jones DR, Lau J, et al Recommendations for examining and interpreting funnel plot asymmetry in meta-analyses of randomised controlled trials. Bmj. 2011;343:d4002 doi: 10.1136/bmj.d4002 21784880 10.1136/bmj.d4002

[25] Deeks JJ, Macaskill P, Irwig L. The performance of tests of publication bias and other sample size effects in systematic reviews of diagnostic test accuracy was assessed. Journal of clinical epidemiology. 2005;58(9):882–93. doi: 10.1016/j.jclinepi.2005.01.016 16085191 10.1016/j.jclinepi.2005.01.016

[26] Sedgwick P. How to read a forest plot in a meta-analysis. Bmj. 2015;351:h4028 doi: 10.1136/bmj.h4028 26208517 10.1136/bmj.h4028

[27] Moses LE, Shapiro D, Littenberg B. Combining independent studies of a diagnostic test into a summary ROC curve: data-analytic approaches and some additional considerations. Statistics in medicine. 1993;12(14):1293–316. 8210827 10.1002/sim.4780121403

[28] Walter SD. Properties of the summary receiver operating characteristic (SROC) curve for diagnostic test data. Statistics in medicine. 2002;21(9):1237–56. doi: 10.1002/sim.1099 12111876 10.1002/sim.1099

[29] Higgins JP, Thompson SG, Deeks JJ, Altman DG. Measuring inconsistency in meta-analyses. Bmj. 2003;327(7414):557–60. doi: 10.1136/bmj.327.7414.557 12958120 10.1136/bmj.327.7414.557 PMC192859

[30] Julian P T Higgins SG. Cochrane Handbook for Systematic Reviews of Interventions: The Cochrane Collaboration; 2011.

[31] R Development Core Team. R: A language and environment for statistical learning. Vienna, Austria: R Foundation for Statistical Computing; 2010.

[32] Lahteenmaki PM, Arola M, Suominen J, Salmi TT, Andersson AM, Toppari J. Male reproductive health after childhood cancer. Acta Paediatr. 2008;97(7):935–42. doi: 10.1111/j.1651-2227.2008.00784.x 18430073 10.1111/j.1651-2227.2008.00784.x

[33] Rendtorff R, Beyer M, Muller A, Dittrich R, Hohmann C, Keil T, et al Low inhibin B levels alone are not a reliable marker of dysfunctional spermatogenesis in childhood cancer survivors. Andrologia. 2012;44 Suppl 1:219–25. doi: 10.1111/j.1439-0272.2011.01167.x 21726269 10.1111/j.1439-0272.2011.01167.x

[34] Bossuyt PM, Reitsma JB, Bruns DE, Gatsonis CA, Glasziou PP, Irwig L, et al STARD 2015: An Updated List of Essential Items for Reporting Diagnostic Accuracy Studies. Clin Chem. 2015;61(12):1446–52. doi: 10.1373/clinchem.2015.246280 26510957 10.1373/clinchem.2015.246280

[35] Whiting PF, Rutjes AW, Westwood ME, Mallett S, Deeks JJ, Reitsma JB, et al QUADAS-2: a revised tool for the quality assessment of diagnostic accuracy studies. Annals of internal medicine. 2011;155(8):529–36. doi: 10.7326/0003-4819-155-8-201110180-00009 22007046 10.7326/0003-4819-155-8-201110180-00009

[36] Hanley JA, McNeil BJ. The meaning and use of the area under a receiver operating characteristic (ROC) curve. Radiology. 1982;143(1):29–36. doi: 10.1148/radiology.143.1.7063747 7063747 10.1148/radiology.143.1.7063747

[37] Makanji Y, Zhu J, Mishra R, Holmquist C, Wong WP, Schwartz NB, et al Inhibin at 90: from discovery to clinical application, a historical review. Endocrine reviews. 2014;35(5):747–94. doi: 10.1210/er.2014-1003 25051334 10.1210/er.2014-1003 PMC4167436

[38] Shin T, Kobayashi T, Shimomura Y, Iwahata T, Suzuki K, Tanaka T, et al Microdissection testicular sperm extraction in Japanese patients with persistent azoospermia after chemotherapy. Int J Clin Oncol. 2016;21(6):1167–71. doi: 10.1007/s10147-016-0998-5 27306218 10.1007/s10147-016-0998-5

[39] Wilhelmsson M, Vatanen A, Borgstrom B, Gustafsson B, Taskinen M, Saarinen-Pihkala UM, et al Adult testicular volume predicts spermatogenetic recovery after allogeneic HSCT in childhood and adolescence. Pediatric blood & cancer. 2014;61(6):1094–100. doi: 10.1002/pbc.24970 24851267 10.1002/pbc.24970

[40] Roberts SC, Seav SM, McDade TW, Dominick SA, Gorman JR, Whitcomb BW, et al Self-collected dried blood spots as a tool for measuring ovarian reserve in young female cancer survivors. Human reproduction. 2016;31(7):1570–8. doi: 10.1093/humrep/dew114 27170433 10.1093/humrep/dew114 PMC4901885

