# Supplementary material for: Follicle Stimulating Hormone is an accurate predictor of azoospermia in childhood cancer survivors
Source: PLoS One. 2017 Jul 20;12(7):e0181377. doi: 10.1371/journal.pone.0181377 (PMC5519149; doi:10.1371/journal.pone.0181377)
Supplement: S1 References — (PDF) [file pone.0181377.s004.pdf]

Follicle Stimulating Hormone is an accurate predictor of azoospermia in childhood cancer survivors

Tom Kelsey
School of Computer Science
University of St Andrews
KY16 9SX, United Kingdom

Lauren McConville
School of Medicine
University of Edinburgh
Edinburgh, United Kingdom

April 30, 2017

Abstract

This is the Supplementary Information file, listing the full-text excluded articles together with reasons for exclusion.

1 WHO

Full texts excluded for non-specification of WHO guidelines for assessment of azoospermia: [Uhler2003, Schrader1998, Pelliccione2011, Kreuser1987, Ortin1990, Bohlen2001, Kader1991, Fossa1980, Kreuser1992, Muller1996, Shamberger1981, Dhabhar1993, BenArush2000]

2 Other Exclusion Ctiteria

Full texts excluded due to data not being reported as scatterplot or box-whisker chart, or for ambiguous demarcation between controls and childhood cancer survivors, or both: [Ishikawa2004, Humpl1999, Kliesch1997, Bohlen2001, Heikens1996, Shamberger1981, LopezAndreu2000, BenArush2000, Wilhelmsson2014, Brydoy2012, Muller1996, Dhabhar1993, Sieniawski2008, Romerius2011]

References

[BenArush2000] M W Ben Arush, I Solt, A Lightman, S Linn, and A Kuten. Male gonadal function in survivors of childhood Hodgkin and non-Hodgkin lymphoma. *Pediatric Hematology & Oncology*, 17(3):239–245, 2000.

Abstract: The aim of this study was to investigate the impact of therapy on long-term gonadal function of young people cured of childhood lymphomas and to assess whether a prepubertal state during the treatment protects the gonads from chemotherapy and/or radiotherapy late effects. Clinical evaluation, semen analysis, and endocrine status were studied in 20 survivors of childhood lymphomas. Five patients received Inverted Y radiotherapy, 2320 cGy (1550-4000); all 20 received chemotherapy as follows: MOPP/ABVD protocol, 9 patients; COMP protocol, 5 patients; MOPP protocol, 3 patients; other protocols, 3 patients. Semen analysis results were as follows: normal values, 4/20 patients; oligospermia, 8/20 patients; azoospermia, 8/20 patients; FSH above normal level, 10/20 patients; 4/5 who received Inverted Y irradiation were azoospermic and 1 was severely oligospermic. Treatment damage to the testis involves tubular germinal elements. Radiotherapy and chemotherapy combinations that included nitrogen mustard or cyclophosphamide were associated with high rates of oligospermia and azoospermia. MOPP/ABVD combination did not have a significant better outcome of sperm counts compared to MOPP alone. Age at chemotherapy did not correlate with the sperm count; hence a prepubertal state did not protect the gonad from the late effects of treatment.

[Bohlen2001] D Bohlen, F C Burkhard, R Mills, R W Sonntag, and U E Studer. Fertility and sexual function following orchiectomy and 2 cycles of chemotherapy for stage I high risk nonseminomatous germ cell cancer. *Journal of Urology*, 165(2):441–444, 2001.

Abstract: PURPOSE: We investigate fertility and sexual function in patients following orchiectomy and adjuvant cisplatin based chemother-

apy for high risk, stage I nonseminomatous germ cell tumor of the testis. MATERIALS AND METHODS: Between 1985 and 1994, 59 patients with stage I nonseminomatous germ cell tumor and poor prognostic factors were treated with 2 cycles of cisplatin, vinblastine and bleomycin, or bleomycin, etoposide and cisplatin after orchiectomy. At least 32 months following treatment all patients were contacted and asked to complete a questionnaire regarding fertility and sexual activity, and to volunteer for a semen and hormonal analysis. RESULTS: Of the 59 patients 49 (83%) completed the questionnaire. Before chemotherapy 18 (37%) patients had fathered children, 6 (12%) were involuntarily childless and none had a major sexual dysfunction. After treatment 11 (22%) patients fathered children, and 5 (10%) were involuntarily childless, with 4 involuntarily childless before chemotherapy. There were no significant alterations in sexual function. Semen analysis in 27 patients was normal in 23, and revealed mild oligospermia in 2 and azoospermia in 2. In 18 patients with hormone analysis median values for luteinizing hormone and free testosterone were normal but median value for follicle-stimulating hormone was slightly increased. CONCLUSIONS: Two cycles of cisplatin based adjuvant chemotherapy do not seem to affect adversely fertility or sexual activity.

[Brydoy2012] M Brydoy, S D Fossa, O Klepp, R M Bremnes, E A Wist, T Bjoro, T Wentzel-Larsen, O Dahl, and I I I study group Norwegian Urology Cancer Group. Sperm counts and endocrinological markers of spermatogenesis in long-term survivors of testicular cancer. *British Journal of Cancer*, 107(11):1833–1839, 2012.

Abstract: BACKGROUND: The objective of this study was to assess markers of spermatogenesis in long-term survivors of testicular cancer (TC) according to treatment, and to explore correlations between the markers and associations with achieved paternity following TC treatment. METHODS: In 1191 TC survivors diagnosed between 1980 and 1994, serum-follicle stimulating hormone (s-FSH; n=1191), s-inhibin

B (n=441), and sperm counts (millions per ml; n=342) were analysed in a national follow-up study in 1998-2002. Paternity was assessed by a questionnaire. RESULTS: At median 11 years follow-up, 44% had oligo- (<15 millions per ml; 29%) or azoospermia (15%). Sperm counts and s-inhibin B were significantly lower and s-FSH was higher after chemotherapy, but not after radiotherapy (RT), when compared with surgery only. All measures were significantly more abnormal following high doses of chemotherapy (cisplatin (Cis)>850 mg, absolute cumulative dose) compared with lower doses (Cis < 850 mg). Sperm counts were moderately correlated with s-FSH (-0.500), s-inhibin B (0.455), and s-inhibin B : FSH ratio (-0.524; all P<0.001). All markers differed significantly between those who had achieved post-treatment fatherhood and those with unsuccessful attempts. CONCLUSION: The RT had no long-term effects on the assessed markers of spermatogenesis, whereas chemotherapy had. At present, the routine evaluation of s-inhibin B adds little in the initial fertility evaluation of TC survivors.

[Dhabhar1993] B N Dhabhar, H Malhotra, R Joseph, S Garde, S Bhasin, A Sheth, and S H Advani. Gonadal function in prepubertal boys following treatment for Hodgkin's disease. *American Journal of Pediatric Hematology/Oncology*, 15(3):306–310, 1993.

Abstract: PURPOSE: Gonadal functions were evaluated in 26 male patients with Hodgkin's disease (HD), who were in continuous unmaintained remission following combination chemotherapy consisting of COPP/MOPP. MATERIALS AND METHODS: These patients had received chemotherapy during the prepubertal phase. The median duration after termination of chemotherapy was 72 months. RESULTS: Semen analysis of 18 patients showed azoospermia. Hormonal analysis showed elevated mean levels follicle-stimulating hormone (FSH) and inhibin as compared to age-matched controls, whereas luteinizing hormone levels were only marginally elevated. CONCLUSIONS: These results suggest that COPP/MOPP causes severe

damage to germinal epithelium even when given during prepubertal age. Sertoli cells, which are responsible for secretion of inhibin, are resistant to these cytotoxic agents. Our data emphasize the lack of gross dysfunction of Leydig cells. It is possible that an alternative chemotherapy protocol (ABVD) may be used in young patients to minimize the gonadal damage.

[Fossa1980] S D Fossa, O Klepp, A Aakvaag, and K Molne. Testicular function after combined chemotherapy for metastatic testicular cancer. *International Journal of Andrology*, 3(1):59–65, 1980.

Abstract: In 10 patients cured for metastatic testicular cancer by combination chemotherapy serum hormone levels and serum agglutinating antibodies were analysed 12 to 35 months after discontinuation of the treatment. Together with these examinations sperm analysis was done. All patients had increased levels of follicle stimulating hormone (FSH). Serum testosterone was usually in the lower part of the normal range (below 20 nmol/l). In eight patients the serum agglutinating antibodies were normal, while two patients had increased levels. In all patients azoospermia was observed, indicating long-lasting infertility in patients testicular cancer treated by combination chemotherapy. The possible importance of cryopreservation prior to start of chemotherapy is discussed.

[Heikens1996] J Heikens, H Behrendt, R Adriaanse, and A Berghout. Irreversible gonadal damage in male survivors of pediatric Hodgkin's disease. *Cancer*, 78(9):2020–2024, 1996.

Abstract: BACKGROUND: Gonadal damage in adult patients after chemotherapy for Hodgkin's disease is well documented, but data of patients treated before adulthood are scarce. METHODS: Gonadal and hormonal function were studied in 19 male long term survivors of Hodgkin's disease who were treated with mechlorethamine, vincristine, procarbazine, and prednisone (MOPP chemotherapy) before (n = 15) or during puberty (n = 4). The studies were performed a median of 10 years after treatment and repeated in the

majority of the patients at the time of yearly visits. RESULTS: Germ cell damage was present in all patients. Semen analysis revealed azoospermia in 12 patients and oligospermia in 6; no recovery of spermatogenesis was seen at follow-up. Testicular size was small in all but one patient. Follicle-stimulating hormone levels were elevated (mean, 14.4 +/- 7.8 U/l) and increased over time (mean, 21.1 +/- 10.5 U/l, $P < 0.001$). In seven patients, luteinizing hormone (LH) was elevated, indicating Leydig cell dysfunction; also in four of those patients, plasma testosterone was decreased. In three other patients, the response of LH to gonadotropin-releasing hormone was exaggerated with a normal basal LH and testosterone. Comparing testicular function of prepubescent versus pubescent state at time of treatment appears to show a trend for improved outcome in the younger patients. CONCLUSIONS: Gonadal function of long term survivors of pediatric Hodgkin's disease treated with MOPP chemotherapy is severely impaired permanently.

[Humpl1999] T Humpl, P Schramm, and P Gutjahr. Male fertility in long-term survivors of childhood ALL. *Archives of Andrology*, 43(2):123–129, 1999.

Abstract: A study of fertility was conducted in postpubertal male patients who had been treated for acute lymphoblastic leukemia (ALL) during childhood or adolescence between 1970 and 1980. Thirteen men (age 18 to 35 years) participated on a volunteer basis. Their age at diagnosis was between 2 and 15 years. Therapy followed the protocol "Memphis VII (Pinkel)." Interview, physical examination, andrological studies (ejaculate), and hormone status (luteinizing hormone, follicle-stimulating hormone, and testosterone) were performed at least 5 years after completion of therapy. No normozoospermia was achieved; 10 patients were identified with asthenozoospermia and 3 patients with azoospermia. With respect to these data, patients treated for ALL between 1970 and 1980 have more significantly impaired spermatogenesis than expected.

[Ishikawa2004] T Ishikawa, S Kamidono, and M Fujisawa. Fertility after high-dose chemotherapy for testicular cancer. *Urology*, 63(1):137–140, 2004.

Abstract: OBJECTIVES: To describe long-term gonadal function after high-dose chemotherapy (HDC). HDC for testicular cancer was recently developed. The evaluation of testicular function after chemotherapy for testicular cancer is an important part of overall care, especially in young patients. METHODS: Between 1994 and 2001, 27 patients underwent HDC (1250 mg/m2 carboplatin, 1500 mg/m2 etoposide, and 7.5 g/m2 ifosfamide) at Kobe University Hospital. Information on gonadal function during follow-up was available for 10 of these patients. The mean patient age +/- SD at treatment was 32.2 +/- 8.4 years. The relationships among age at treatment, semen analysis, serum hormone levels (follicle-stimulating hormone, luteinizing hormone, testosterone, prolactin, and estradiol), cumulative dose of cisplatin and carboplatin, and length of follow-up were determined. RESULTS: Spermatogenesis recovered after cessation of HDC in 5 of 10 patients. Semen analysis in these patients showed the mean sperm concentration and motility at 42.4 +/- 10.4 million/mL and 67.2% +/- 17.0%, respectively. The patients were divided into azoospermic and nonazoospermic groups. The age of the nonazoospermic and azoospermic patients was 28.2 +/- 8.7 and 36.2 +/- 6.5 years, respectively. Follicle-stimulating hormone levels in the nonazoospermic group (11.7 +/- 3.4 mIU/mL) were significantly lower than in the azoospermic group (32.8 +/- 14.4 mIU/mL; P = 0.0472). No other statistically significant difference was observed in the other hormone levels or the cumulative dose of cisplatin and carboplatin between the azoospermic and nonazoospermic groups. CONCLUSIONS: Spermatogenesis recovers after HDC in some patients. Patients should be informed that they may or may not be fertile after HDC.

[Kader1991] H A Kader and A Y Rostom. Follicle stimulating hormone levels as a predictor of recovery of spermatogenesis fol-

lowing cancer therapy. *Clinical Oncology (Royal College of Radiologists)*, 3(1):37–40, 1991.

Abstract: Infertility, both temporary and permanent, is a well-recognized complication of certain cancer treatments. The main objective of this study was to determine whether recovery of fertility in male patients, could be predicted by monitoring changes in serum follicle stimulating hormone (FSH) levels. Twenty male patients participated in the study. Sperm counts and serum FSH levels were measured before, during and after treatment. Azoospermia was universal in all 20 patients during the first year, with significantly raised FSH in almost all the patients. Reduction of FSH levels during the second year was frequently followed by recovery of spermatogenesis. Patients in whom the FSH did not fall during the second year were highly unlikely to regain fertility.

[Kliesch1997] S Kliesch, M Bergmann, L Hertle, E Nieschlag, and H M Behre. Semen parameters and testicular pathology in men with testicular cancer and contralateral carcinoma in situ or bilateral testicular malignancies. *Human Reproduction*, 12(12):2830–2835, 1997.

Abstract: We evaluated 14 patients with bilateral testicular tumour, one-sided tumour and contralateral carcinoma in situ (CIS) of the testis or testis tumour in single testis with respect to their fertility. We analysed semen parameters, serum hormones [follicle-stimulating hormone (FSH), luteinizing hormone (LH) and testosterone], testicular sonography, testicular volumes and testicular histology prior to further anti-cancer treatment. Ten out of 14 patients showed normal or reduced sperm concentrations, while 4/14 patients were azoospermic. Serum FSH levels showed a significant negative correlation with sperm concentrations in patients with testicular malignancies ($r = -0.64$, $P = 0.025$). Testicular volumes revealed a significant positive correlation with semen parameters in patients with testes that were affected by CIS ($r = 0.733$, $P = 0.038$). We conclude that even bilateral testicular cancer and/or CIS do not pre-

clude fertility and, therefore, patients should be offered andrological investigation and therapy, including possibly surveillance strategy or the chance for cryopreservation of the semen prior to further treatment in order to preserve their chances for paternity.

[Kreuser1987] E D Kreuser, N Xiros, W D Hetzel, and H Heimpel. Reproductive and endocrine gonadal capacity in patients treated with COPP chemotherapy for Hodgkin's disease. *Journal of Cancer Research & Clinical Oncology*, 113(3):260–266, 1987.

Abstract: Testicular and ovarian functions were assessed in 33 patients with Hodgkin's disease 1 to 17 years after cessation of COPP chemotherapy with cyclophosphamide, vincristine, procarbazine, prednisone. Diagnostic procedures consisted of hormone measurements, interviews, and semen analyses. In women serum levels of follicle-stimulating hormone (FSH), luteinizing hormone (LH), 17 beta-estradiol, progesterone, prolactin, and in men FSH, LH, 17 beta-estradiol, testosterone, and prolactin were determined. Semen analyses were performed in all men. Information concerning pregnancies, pregnancy outcome, future fertility wishes, sexual functions, menstrual pattern, and incidence of premature menopausal symptoms was ascertained by interview and questionnaire. Nineteen of 19 (100%) men showed elevated serum FSH levels between 715 and 1910 (median 1095) ng/ml and azoospermia, 1 to 11 years after therapy. Serum levels of testosterone were within normal limits in 18/19 (95%) of the men, and LH values were normal in all men. Permanent ovarian failure occurred in 8/14 (57%) women, causing infertility and premature menopausal symptoms. The incidence of ovarian failure in women over 24 years was 86% (6/7) versus 28% (2/7) in those under 24 years at the time of treatment. In women receiving estrogen replacement, incidence and severity of these symptoms were significantly reduced. Of 14 women 3 (21%) became pregnant and delivered 5 healthy children after treatment. Our results suggest irreversible sterility and normal Leydig cell function after

COPP chemotherapy in all men. Drug-induced ovarian failure was age-related and caused premature menopausal symptoms, detracting from the quality of the patient's life. To reduce premature menopausal symptoms and to prevent adverse cardiovascular and metabolic late sequelae, hormonal replacement is indicated. Pregnancies ending in normal live births can be achieved after COPP chemotherapy in young women. In both men and women, serum FSH and LH levels proved to be feasible markers to determine degree and duration of endocrine and reproductive gonadal injury after chemotherapy.

[Kreuser1992] E D Kreuser, D Felsenberg, C Behles, H Seibt-Jung, M Mielcarek, V Diehl, E Dahmen, and E Thiel. Long-term gonadal dysfunction and its impact on bone mineralization in patients following COPP/ABVD chemotherapy for Hodgkin's disease. *Annals of Oncology*, 3 Suppl 4:105–110, 1992.

Abstract: Only limited data is currently available on long-term gonadal toxicity and its impact on bone mineralization in men and women treated for Hodgkin's disease. The present study was therefore conducted to evaluate gonadal toxicity and bone loss in 49 patients with Hodgkin's disease 2-10 (median 5.37) years after chemotherapy. Most patients were treated with the COPP/ABVD regimen +/- irradiation according to the protocols of the German Hodgkin Study Group. Blood samples were tested for gonadotropins (FSH, LH), gonadal steroids, parathyroid hormone, osteocalcin, and calcitonin. Bone mineral density was measured using single- and dual-energy quantitative computed tomography as well as single-photon absorptiometry. FSH serum levels were significantly increased in 21/27 (80%) men demonstrating germ-cell aplasia. 13/15 (86%) men showed azoospermia after the COPP/ABVD regimen. In contrast, testosterone levels were within normal limits in all men tested, suggesting normal Leydig-cell function. 17/22 (77%) women exhibited increased FSH and LH levels, indicating premature ovarian failure. Women with

therapy-induced ovarian failure had a significantly lower trabecular (98 +/- 34) and cortical (292 +/- 48 mg/cm3) spinal bone density than those with normal ovarian function. Men showed no evidence of bone loss after therapy. These data suggest severe gonadal toxicity in both men and women treated with the COPP/ABVD regimen. In female patients, drug-induced ovarian failure has a significant impact on bone mineralization.

[LopezAndreu2000] J A Lopez Andreu, P J Fernandez, J Ferris i Tortajada, I Navarro, A Rodriguez-Ineba, P Antonio, M D Muro, and A Romeu. Persistent altered spermatogenesis in long-term childhood cancer survivors. *Pediatric Hematology & Oncology*, 17(1):21–30, 2000.

Abstract: This study evaluated male gonadal function in long-term survivors of childhood cancer and assessed the suitability of offering sperm analysis to all those patients independently of the diagnosis and treatment received. A total of 43 survivors of acute lymphoblastic leukemia (21), acute myeloid leukemia (1), neuroblastoma (8), ganglioneuroblastoma (1), ganglioneuroma (2), Wilms' tumor (9), and mesoblastic nephroma (1) underwent sperm analysis at a mean age of 20.2 years, after a mean time off treatment of 13.6 years. Eight of the patients (19%) were azoospermic, 2 (5%) were severely oligo-asthenozoospermic, and only 16 (37%) were normozoospermic. A control group of healthy volunteers aged < or = 30 years included no azoospermic subjects, 7% severely oligo-asthenozoospermic, and 67% normozoospermic. Comparisons were also made with patients treated at our Human Reproductive Unit aged < or = 30 years (n = 373) whose percentages for the above parameters were 4, 9, and 42%, respectively. Cumulated cyclophosphamide dose and basal follicle-stimulating hormone (FSH) levels were identified as independent factors associated with azoospermia or severe oligo-asthenozoospermia. Azoospermic and severely oligo-asthenozoospermic survivors had significantly smaller mean testicular volume and higher basal FSH levels than the other survivors,

but small testicles (sum of both testicular volume < or = 20 mL) and/or abnormally high basal FSH (> 10 mIU/mL) were present in only half of the azoospermic survivors. Male long-term survivors of childhood cancer constitute a high-risk subpopulation for altered sperm analysis. It seems justified to offer sperm analysis to all long-term survivors.

[Muller1996] H L Muller, M Klinkhammer-Schalke, B Seelbach-Gobel, A A Hartmann, and J Kuhl. Gonadal function of young adults after therapy of malignancies during childhood or adolescence. *European Journal of Pediatrics*, 155(9):763–769, 1996.

Abstract: UNLABELLED: As the survival rate of children with malignancies has increased over past decades, the follow up of adult long-term survivors (LTS) of childhood cancer should focus on late effects of disease and treatment. Gonadal function was therefore studied in 54 LTS (aged 17-29 years; 33 male, 21 female) 2-18 years after treatment for malignancies during childhood or adolescence. To analyse the sensitivity of different diagnostic methods, tests of endocrine function (n = 52), spermiograms (n = 14), gynaecological status (n = 20) and ultrasonography of the gonads (n = 53) were compared with the results of equivalent tests in 23 age-matched normal controls (12 male, 11 female). There were no differences between male and female LTS concerning age at diagnosis, gonadal dose of irradiation (XRT) and doses of applied chemotherapeutic agents. Whereas male LTS had elevated levels of luteinizing hormone (LH) and follicle-stimulating hormone (FSH) before (P < 0.05; P < 0.001) and after (P < 0.01; P < 0.001) stimulation with gonadotropin releasing hormone, female LTS exhibited normal endocrine function. Accordingly, male patients exhibited lower testicular volumes than normal controls, as measured with a Prader orchidometer (P < 0.01) or by ultrasonography (P < 0.001). Gynaecological status and ultrasonography of the gonads were normal in female LTS and controls. Whereas all spermiograms of normal controls (n = 8) showed a normal sperm cell density (SCD), only 2 of 14

male LTS exhibited a normal SCD ($P < 0.001$). Azoospermic LTS ($n = 9$) had been treated more often with alkylating agents and had received higher ($P < 0.05$) gonadal doses of XRT. All male LTS with testicular volumes below the normal range (< 13 ml) and basal FSH levels above the normal range (> 10 IU/l) exhibited azoospermia, whereas LTS with normal values for testicular volume and basal FSH had a normal SCD. CONCLUSION: A sex-specific susceptibility for gonadal damage after treatment for malignancies might be responsible, in part, for the impaired gonadal function of male LTS. Therapy with alkylating agents and/or high gonadal doses of XRT were important risk factors for azoospermia. A simple method to estimate potential fertility in individual LTS is to measure testicular volume, using a Prader orchidometer, and basal FSH serum levels.

[Ortin1990] T T Ortin, C A Shostak, and S S Donaldson. Gonadal status and reproductive function following treatment for Hodgkin's disease in childhood: the Stanford experience. *International Journal of Radiation Oncology, Biology, Physics*, 19(4):873–880, 1990.

Abstract: To ascertain the impact of therapy on gonadal function and reproductive outcome among children treated for Hodgkin's disease, we reviewed the experience at Stanford University Medical Center during the years 1965-1986. There were 240 children 15 years of age or younger, 92 girls and 148 boys; with median follow-up of 9 years, maximum follow-up was 26 years. Of this cohort, data on gonadal function were available on 20 boys, 5 of whom were considered prepubescent; they had no clinical evidence of sexual maturation and were less than 13 years of age. Evaluation of the boys included testicular biopsy, semen analyses and the ability to procreate. Serum gonadotropin hormone levels (FSH, LH) were studied in 11 boys who also had semen analyses. Sexual maturation was attained in all boys without the need for androgen replacement. Among the eight boys treated with radiation alone, four were able to father a child (3 following 40-45 Gy pelvic radiation dose,

1 without pelvic radiation) from 3-19 years following treatment. Three others who received 30-44 Gy pelvic radiation were oligospermic when tested at 10 to 15 years post-treatment. Semen analyses in 10 of 12 (83%) boys who had been treated with six cycles of MOPP with or without pelvic radiation revealed absolute azoospermia with no evidence of recovery as along as 11 years of follow-up. Following prolonged azoospermia, 2 of the 12 boys (17%) had recovery of fertility, with normalization of sperm count and/or ability to procreate at 12 and 15 years following treatment. There was no correlation with serum gonadotropin levels and sterility. Data on menstrual history, pregnancy and offspring were available in 86 (92%) of the girls. Seventy-five of the 86 girls (87%) have normal menstrual function. However, none of the females who underwent pelvic radiation without prior oophoropexy has maintained ovarian function. Both the prepubescent and postpubescent boys were affected by 6 cycles of MOPP whether or not pelvic radiation was administered. On the other hand, in girls similarly treated, ovarian injury was directly related to both the number of cycles of chemotherapy and the ovarian radiation dose. The chances of maintaining gonadal function following combined modality treatment are significantly greater among girls than boys. The progeny of patients treated for Hodgkin's disease appear normal and no excess fetal wastage has been noted.

[Pelliccione2011] F Pelliccione, V Verratti, A D'Angeli, A Micillo, C Doria, A Pezzella, G Iacutone, F Francavilla, C Di Giulio, and S Francavilla. Physical exercise at high altitude is associated with a testicular dysfunction leading to reduced sperm concentration but healthy sperm quality. *Fertility & Sterility*, 96(1):28–33, 2011.

Abstract: OBJECTIVE: To explore the effect of physical exercise at high altitudes (HA) on male reproductive system. DESIGN: Prospective study. SETTING: Andrology Clinic, University of L'Aquila, Italy. PATIENT(S): Seven male mountaineers involved in an expedition at 5,900 m. INTERVENTION(S): Semen analysis, sperm DNA

fragmentation with flow cytometry, and reproductive hormone levels. MAIN OUTCOME MEASURE(S): Hormone levels were evaluated at sea level (SL) at baseline (SL-pre), after 22 days of exercise at HA (intermediate), and after 10 days upon reaching SL (SL-post). Sperm parameters, percentage of sperm with fragmented DNA, and body composition measures were evaluated at SL-pre and at SL-post. RESULT(S): A reduction of sperm concentration, of body mass index (BMI), of waist circumference, and of percentage of body fat was observed at SL-post compared with SL-pre values. Increased levels of FSH and PRL were observed at the intermediate point, and normalized at SL-post, whereas T was higher at SL-post compared with SL-pre levels. CONCLUSION(S): Physical exercise at HA is associated with a testicular dysfunction leading to a reduced sperm concentration probably through an altered spermiation. The improved body composition after physical exercise might explain the higher T levels observed after the expedition.Copyright © 2011 American Society for Reproductive Medicine. Published by Elsevier Inc. All rights reserved.

[Romerius2011] P Romerius, O Stahl, C Moell, T Relander, E Cavallin-Stahl, T Wiebe, Y L Giwercman, and A Giwercman. High risk of azoospermia in men treated for childhood cancer. *International Journal of Andrology*, 34(1):69–76, 2011.

Abstract: Childhood cancer survivors (CCS) have an increased risk of impaired spermatogenesis, but data regarding the disease- and treatment-related risk factors of azoospermia are scarce. Such information is crucial both for counselling CCS and for selecting patients for testicular tissue cryopreservation. The proportion of azoospermic men in CCS was 18% [95% confidence interval (CI): 12-26], specifically for leukaemias (19%; 95% CI: 5.5-42), Hodgkin's disease (53%; 95% CI: 29-76), non-Hodgkin's lymphoma (11%; 95% CI: 0.28-48) and testicular cancer (11%; 95% CI: 0.28-48). In CCS treated with high doses of alkylating agents, the proportion of azoospermic men was 80% (95% CI: 28-99) and if radiotherapy was used additionally,

the proportion was 64% (95% CI: 35-87). In CCS with subnormal Inhibin B levels, the proportion of azoospermic men was 66% (95% CI: 47-81) and for those with elevated follicle-stimulating hormone (FSH) levels, the proportion was 50% (95% CI: 35-67). Among CCS with subnormal testicular volume (< 24 mL), azoospermia was found in 61% (95% CI: 39-80) of the cases. Most childhood cancer diagnoses are associated with an increased risk of azoospermia, especially in CCS receiving testicular irradiation, high doses of alkylating drugs and other types of cytotoxic treatment, if combined with irradiation. Inhibin B, FSH and testicular volume can be used as predictors for the risk of azoospermia.Copyright © 2010 The Authors. International Journal of Andrology © 2010 European Academy of Andrology.

[Schrader1998] S M Schrader, R E Langford, T W Turner, M J Breitenstein, J C Clark, B L Jenkins, D O Lundy, S D Simon, and T B Weyandt. Reproductive function in relation to duty assignments among military personnel. *Reproductive Toxicology*, 12(4):465–468, 1998.

Abstract: As a follow-up to the pilot study of semen quality of soldiers with various military assignments a larger, more complete study was conducted. Soldiers were recruited at Fort Hood, Texas. Thirty-three men were exposed to radar as part of their duty assignment in the Signal Corps, 57 men were involved with firing the 155 mm howitzer (potential lead exposure), and 103 soldiers had neither lead nor radar exposure and served as the comparison control group. Both serum and urinary follicle-stimulating hormone and luteinizing hormone and serum, salivary, and urine testosterone levels were determined in all men. A complete semen analysis was conducted on each soldier. For statistical analysis, the primary study variables were: sperm concentration, sperm/ejaculate, semen volume, percent normal morphology, percent motile, percent viable (both vital stain and hypoosmotic swelling), curvilinear velocity, straight-line velocity, linearity, sperm head length, width, area,

and perimeter. Variables were adjusted for significant confounders (e.g., abstinence, sample age, race). No statistical differences ($P < 0.05$) were observed in any measurement. While these results are in agreement with two previous studies assessing soldiers firing the 155-mm howitzer, they contradict our previous report indicating that radar exposure caused a significant decrease in sperm numbers. A possible explanation is that the radar exposure in this study was that used in Signal Corps operations while the men in the previous study were using different radar as part of military intelligence operations. The data presented here in men firing the 155-mm howitzer combined with the results from the previous studies confirms that there are no deficits in semen quality in these men. The contradiction between the results of the radar exposure studies indicates that more data are needed to evaluate the relationship of military radar and male reproductive health.

[Shamberger1981] R C Shamberger, R J Sherins, and S A Rosenberg. The effects of postoperative adjuvant chemotherapy and radiotherapy on testicular function in men undergoing treatment for soft tissue sarcoma. *Cancer*, 47(10):2368–2374, 1981.

Abstract: Testicular function was studied in 26 men with sarcoma who received adjuvant treatment with doxorubicin, cyclophosphamide, and high-dose methotrexate (with or without radiotherapy). Testicular size, sperm output, and serum FSH, LH and testosterone levels were assessed after treatment. Five of 17 men who received chemotherapy or chemotherapy with radiotherapy to the neck, arm, chest, or leg, had normal testicular function. Eight of the remaining 12 men who provided ejaculates were oligospermic or azoospermic and serum FSH was increased threefold and LH twofold; testosterone levels were normal. In the five men with normal testicular function, FSH was increased fourfold during therapy but returned to normal six to 21 months after treatment. In men less than 40 years old, the mean FSH was less than that of men over 40 years of age (P =

to 0.05), suggesting that recovery from the injury was age-related. By contrast, all nine men who received chemotherapy plus radiotherapy to the abdomen or thigh had decreased testicular size, azoospermia, fourfold increase in FSH, and twofold increase in LH levels; but testosterone concentration was normal. These data increase in FSH, and reversible testicular injury occurs after treatment with doxorubicin, cyclophosphamide, and high-dose methotrexate; recovery is age-related. However, these agents in combination with use of adjuvant radiotherapy to the thigh or abdomen may produce permanent testicular injury even in young patients.

[Sieniawski2008] M Sieniawski, T Reineke, A Josting, L Nogova, K Behringer, T Halbsguth, M Fuchs, V Diehl, and A Engert. Assessment of male fertility in patients with Hodgkin's lymphoma treated in the German Hodgkin Study Group (GHSG) clinical trials. *Annals of Oncology*, 19(10):1795–1801, 2008.

Abstract: BACKGROUND: Infertility is one of the most significant side-effects in long-term survivors of successfully treated Hodgkin's lymphoma (HL). PATIENTS AND METHODS: The fertility status was assessed in male HL patients enrolled into trials of the German Hodgkin Study Group from 1988 to 2003. RESULTS: In pre-treatment analysis (n = 202), 20% of patients had normozoospermia, 11% azoospermia and 69% had other dyspermia. In post-treatment analysis (n = 112), 64% of patients had azoospermia, 30% other dyspermia and 6% normozoospermia ($P < 0.001$). Azoospermia was observed in 90% of patients treated with chemotherapy alone, 67% of those treated with combined modality and 11% of those treated with radiotherapy alone ($P < 0.001$). Azoospermia was more frequent after 4x cyclophosphamide, vincristine, procarbazine, prednisone, doxorubicin, bleomycin, vinblastine, dacarbazine (COPP/ABVD) (91%), 8x bleomycin, etoposide, doxorubicin, cyclophosphamide, vincristine, procarbazine, prednisone (BEACOPP) baseline (93%) and 8x BEACOPP escalated (87%) compared with 2x COPP/ABVD (56%; $P = 0.003$).

There was a statistically significant difference in post-treatment follicle-stimulating hormone (FSH) levels between patients with azoospermia and those with preserved spermatogenesis (P = 0.001). CONCLUSIONS: Depending on the treatment received, male HL patients are at high risk of infertility after treatment. FSH might be used as surrogate parameter for male fertility in future studies.

[Uhler2003] M L Uhler, M J Zinaman, C C Brown, and E D Clegg. Relationship between sperm characteristics and hormonal parameters in normal couples. *Fertility & Sterility*, 79 Suppl 3:1535–1542, 2003.

Abstract: OBJECTIVE: To examine the relationships between hormone profiles and semen analysis measures and fertility in the male partners of presumed normal couples. DESIGN: Prospective clinical study. SETTINGS: Healthy volunteers in an academic research environment. PATIENT(S): One hundred forty-five reproductive age couples without known risk factors for infertility and who had discontinued contraception to achieve pregnancy completed this component of this study. Each couple was followed for < or =12 menstrual cycles while they attempted to conceive. INTERVENTION(S): Semen quality measures for the first ejaculates were obtained at the start of the study along with a single blood sample. Levels of FSH, bioactive FSH, inhibin B, LH, and T were measured for each man. MAIN OUTCOME MEASURE(S): Semen analysis, FSH, inhibin B, LH, T, and clinical pregnancy. RESULTS: Significant positive relationships were observed between the two measures of FSH as well as between both of the FSH measures and LH. Follicle-stimulating hormone as measured by RIA was significantly negatively correlated with inhibin B. Inhibin B showed a marginally significant negative correlation with LH, and LH and T had a marginally significant positive correlation. Inhibin B increased significantly, and both measures of FSH activity showed significant decreases, with increasing levels in several semen quality measures. There was no significant relationship between

the measured hormones and the pregnant and nonpregnant groups or time to pregnancy. CONCLUSION(S): These results contribute additional information on the utility of reproductive hormone measurements for predicting semen quality in couples without known reduced fertility.

[Wilhelmsson2014] Mari Wilhelmsson, Anu Vatanen, Birgit Borgström, Britt Gustafsson, Mervi Taskinen, Ulla M. Saarinen-Pihkala, Jacek Winiarski, and Kirsi Jahnukainen. Adult testicular volume predicts spermatogenetic recovery after allogeneic HSCT in childhood and adolescence. *Pediatric Blood & Cancer*, 61(6):1094–1100, jun 2014.
